# Analysis of miRNA rare variants in amyotrophic lateral sclerosis and *in silico* prediction of their biological effects

**DOI:** 10.3389/fgene.2022.1055313

**Published:** 2022-12-07

**Authors:** Alberto Brusati, Antonia Ratti, Viviana Pensato, Silvia Peverelli, Davide Gentilini, Eleonora Dalla Bella, Marta Nice Sorce, Megi Meneri, Delia Gagliardi, Stefania Corti, Cinzia Gellera, Giuseppe Lauria Pinter, Nicola Ticozzi, Vincenzo Silani

**Affiliations:** ^1^ Department of Neurology and Laboratory of Neuroscience, IRCCS Istituto Auxologico Italiano, Milan, Italy; ^2^ Department Brain and Behavioral Sciences, University of Pavia, Pavia, Italy; ^3^ Department Medical Biotechnology and Translational Medicine, University of Milan, Milan, Italy; ^4^ Fondazione IRCCS Istituto Neurologico Carlo Besta, Milano, Italy; ^5^ Bioinformatics and Statistical Genomics Unit,IRCCS Istituto Auxologico Italiano,Milan,Italy; ^6^ Fondazione IRCCS Cà Granda Ospedale Maggiore Policlinico, Milano, Italy; ^7^ Department of Pathophysiology and Transplantation, Dino Ferrari Center, University of Milan, Milan, Italy

**Keywords:** ALS, miRNA, WGS, rare variants, bioinformatics

## Abstract

**Background:** Amyotrophic lateral sclerosis (ALS) is a neurodegenerative disease affecting upper and/or lower motor neurons and characterized by complex etiology. Familial cases show high genetic heterogeneity and sporadic cases (90%) are associated with several genetic and environmental risk factors. Among the genetic risk factors, the contribution of non-coding elements, such as microRNAs (miRNAs), to ALS disease susceptibility remains largely unexplored.

**Aim:** This work aims to identify rare variants in miRNA genes in sporadic ALS (sALS) patients which may cause a defective miRNA maturation or altered target gene recognition by changing miRNA secondary structure or seed sequence, respectively.

**Methods:** Rare variants located in miRNA loci with a minor allele frequency (MAF) < 0.01 were extracted from whole genome sequencing (WGS) data of 100 sALS patients. The secondary pre-miRNA structures were predicted using MiRVas to evaluate the impact of the variants on RNA folding process. Human TargetScan was used to retrieve all the potential target genes of miRNAs with variants in the seed region. Over Representation Analysis (ORA) was conducted to compare the lists of target genes for the reference and mutated miRNAs in the seed sequence.

**Results:** Our analysis identified 86 rare variants in 77 distinct miRNAs and distributed in different parts of the miRNA precursors. The presence of these variants changed miRNA secondary structures in ∼70% of MiRVas predictions. By focusing on the 6 rare variants mapping within the seed sequence, the predicted target genes increased in number compared to the reference miRNA and included novel targets in a proportion ranging from 30 to 82%. Interestingly, ORA revealed significant changes in gene set enrichment only for mutated miR-509-1 and miR-941-3 for which the Gene Ontology term related to “nervous system development” was absent and present, respectively, compared to target lists of the reference miRNA.

**Conclusion:** We here developed a workflow to study miRNA rare variants from WGS data and to predict their biological effects on miRNA folding, maturation and target gene recognition. Although this *in silico* approach certainly needs functional validation *in vitro* and *in vivo*, it may help define the role of miRNA variability in ALS and complex diseases.

## Introduction

Amyotrophic lateral sclerosis (ALS) is a rare adult-onset neurodegenerative disease resulting in the progressive loss of upper and/or lower motor neurons that in turn leads to muscle paralysis and death for respiratory failure usually within 3–5 years after onset. Familial ALS (fALS), which explains only 10% of the forms, is associated with high genetic heterogeneity with more than 30 causative genes involved in different cell pathways (Hardiman et al., 2017). For sporadic ALS (sALS) the etiopathogenesis remains largely unclear with a multifactorial model, involving both genetic and environmental risk factors (Goutman et al., 2022). High-throughput technologies, including genome-wide association studies (GWAS) and epigenome-wide association studies (EWAS), have been recently combined to identify common genetic variants and epigenetic signatures, respectively, which are significantly associated with disease susceptibility (van Rheenen et al., 2021; Hop et al., 2022). Additionally, next-generation sequencing (NGS) technologies significantly boosted the advances in ALS genetics, in particular, coupling the use of whole-exome (WES) and whole-genome (WGS) sequencing with computational methods to identify rare variants associated with both fALS and sALS (Goutman et al., 2022). Despite the identification of novel causative and risk genes, the complexity of sALS genetics remains widely uncharacterized and about 30% of fALS heritability is still missing.

The role of non-coding DNA in complex diseases has been only recently explored. Variants in key regulatory sequences may affect transcription by changing chromatin folding and accessibility as well as post-transcriptional processes, including splicing and mRNA fate, altogether and ultimately affecting gene expression (Spielmann and Mundlos, 2016). MicroRNAs (miRNAs) are a class of small non-coding elements broadly studied for their spatiotemporal control of gene expression in different tissues and specific cell types. Although dysregulation of their levels has been studied in neurodegenerative diseases, including ALS (Juźwik et al., 2019), miRNA genetic variability in patients, as well as the biological effects of miRNA genetic variants are still scarcely studied. In the last 2 decades, only a small number of studies has described the impact of non-coding variants on correct miRNA biogenesis and target gene recognition and expression. In particular, this functional association was described for complex disorders such as rasopathies (de Carvalho et al., 2019), autism (Williams et al., 2019), and schizophrenia (Duan et al., 2014), as well as monogenic disorders such as spondyloepiphyseal dysplasia (Grigelioniene et al., 2019) and nonsyndromic hearing loss (Mencía et al., 2009). In ALS, miRNAs have mainly been investigated as potential biomarkers being dysregulated in patients’ biofluids, brain, and skeletal muscle (Di Pietro et al., 2018; Rinchetti et al., 2018; Joilin et al., 2019; Kim et al., 2020; Alvia et al., 2022; Panio et al., 2022). A single recent study identified, in a large cohort of ALS patients, 6 rare variants in miR-218-2, which were associated with its defective biogenesis and maturation leading to downregulation of the miRNA itself and, consequently, to the upregulation of its target gene expression (Reichenstein et al., 2019).

The aim of our analysis was to identify rare variants in miRNAs from WGS data of a cohort of sALS patients and to predict their biological effects on miRNA secondary structure, maturation, and target gene recognition by bioinformatic tools, providing a general workflow to be used for miRNA analysis in other complex disorders.

## Materials and methods

### Clinical data

A cohort of 100 sALS patients, 44 males and 56 females, was selected for WGS analysis. The median age of onset was 64 years and the age at DNA collection was 65 years (Table 1). The majority of patients (81%) showed a spinal onset of the disease, while 17% and 2% exhibited a bulbar and a respiratory onset, respectively (Table 1). All patients had a diagnosis of ALS based on the revised El Escorial criteria (Brooks et al., 2000). The Ethics Committees of the participating Institutions approved the study. All participants gave written informed consent for using pseudonymized clinical and genetic data for research purposes. The study was performed in accordance with the principles of the Declaration of Helsinki.

**TABLE 1:** Clinical features of the ALS cohort.

Sporadic ALS patients (n)	100
Gender (n)	44 Males | 56 Females
Site of onset (n)	81 Spinal | 17 Bulbar | 2 Pulmonar
Age at onset (median)	64 (Q1 = 55.5; Q3 = 70,5)
Age at collection (median)	65 (Q1 = 57; Q3 = 72)

### Whole-genome and bioinformatics analysis

**FIGURE 1:**
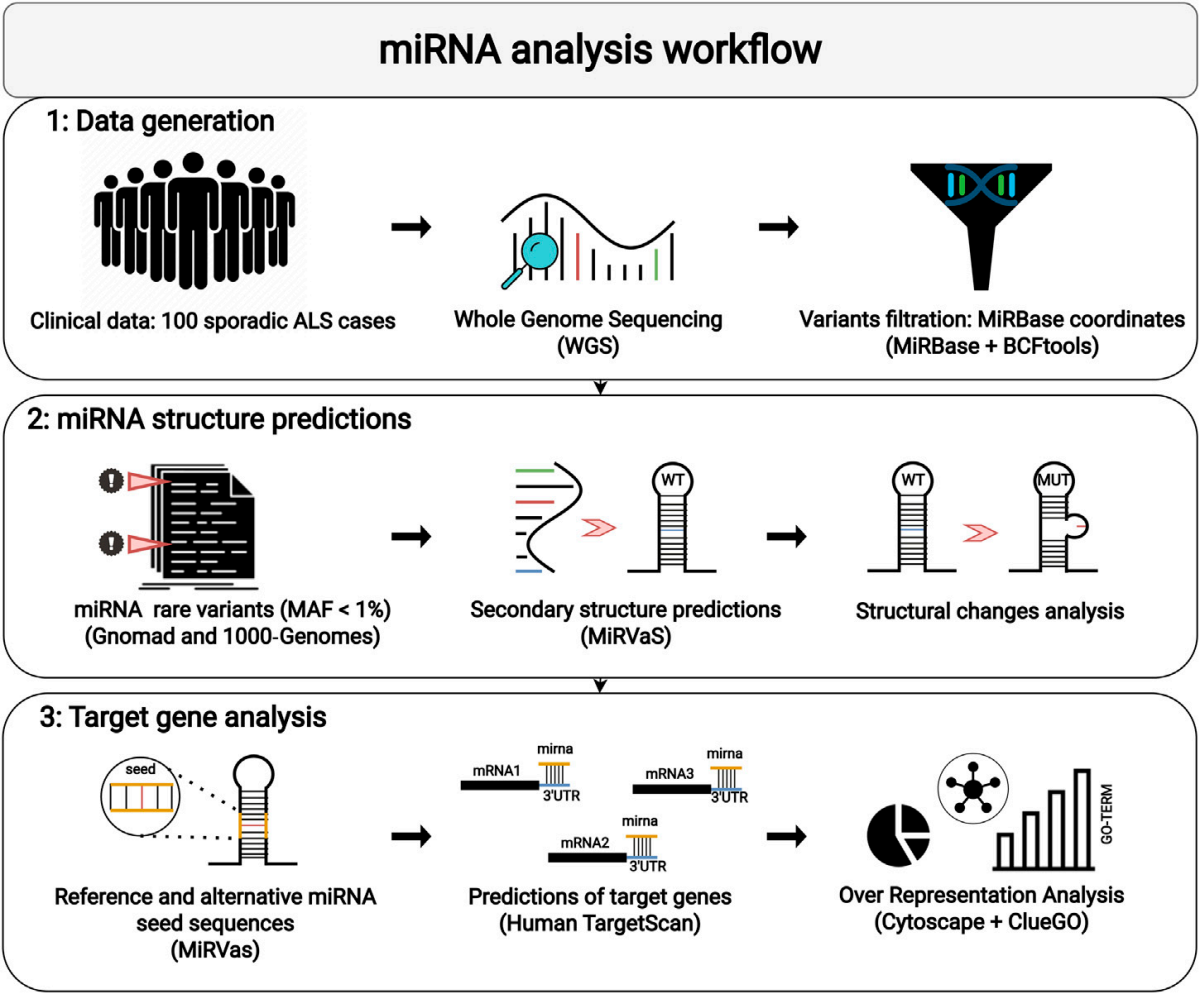
The workflow adopted for miRNA analysis is shown: (1) Selection of sporadic ALS patients for WGS and filtering of rare variants located in miRNAs; (2) Location of variants in miRNA sequences and secondary structures predictions; (3) Target analysis and ORA on reference and alternative miRNA seed sequences.

The pipeline designed for miRNA analysis consisted of three different steps: 1) WGS data generation and filtering, 2) analysis of structural miRNA modifications, and 3) prediction of target genes of miRNAs with variants in the seed sequence (Figure 1). WGS was performed on Illumina NovaSeq platform, with an average coverage yield of ∼50x. Reads were processed according to the best practices pipeline recommended by Broad Institute. Burrows-Wheeler Aligner (BWA) (Li and Durbin, 2009) was employed to align raw data to the reference genome (GRCh37, hg19). The obtained BAM files were deduplicated and recalibrated to add reliability to the final alignment. Genomic data are available at European Nucleotide Archive (ENA; study accession number PRJEB57326). The variant calling step was performed using the GATK tool (McKenna et al., 2010) and HaplotypeCaller (DePristo et al., 2011; Van der Auwera et al., 2013) algorithm. Multiple VCF files were finally assembled *via* VCFtools (Danecek et al., 2011). The annotation of VCF files was completed using ANNOVAR (Wang et al., 2010). After this step, we used the genomic coordinates collected from MiRBase (Kozomara et al., 2019) to retrieve variants included within miRNAs loci considered as “high-confident”. Rare variants were filtered out with a Minor Allele Frequency (MAF) < 0.01 in the population frequency databases 1000 Genomes Project (Devuyst, 2015) and GnomAD (Karczewski et al., 2020). Expression data available for candidate miRNAs were obtained from Genotype-Tissue Expression (GTEx) portal (https://gtexportal.org).

### *In silico* prediction of miRNA secondary structures

We utilized miRVaS (Cammaerts et al., 2016), a tool based on RNAFold (Lorenz et al., 2011) and VARNA (Darty et al., 2009), to evaluate both the location of the identified variant and its possible impact on the secondary structures of the miRNA precursor. Default parameters have been set as recommended, including 100 nucleotides flanking up- and down-stream the pre-miRNA sequence, as derived from functional experiments. Secondary RNA structures were successfully predicted in the presence and the absence of the identified rare variants using three different metrics: 1) minimum free energy (MFE) which presumes a single possible conformation based on minimizing free energy, 2) maximal expected accuracy (MEA) which maximizes the base-pair accuracy using a partition function and, 3) centroid prediction which represents the ensemble of possible secondary structures.

### *In silico* analysis of target genes

Target gene analysis was performed using the Human TargetScan command-line version 8.0 (Agarwal et al., 2015). The seven nucleotides-seed sequence of the reference or the alternative miRNA was matched to TargetScan UTR sequences default database. The obtained Ensembl IDs were assigned to their respective genes with a specifically developed python script. The resulting lists of target genes were subsequently used for Over Representation Analysis (ORA) using ClueGO v.2.5-2021 (Bindea et al., 2009), a CytoScape 3.8 plugin (Shannon et al., 2003). The following criteria were set up for the enrichment analysis: 1) “Biological Processes” was chosen as the primary ontology dataset, 2) GO (Gene Ontology) Term fusion option was selected to reduce the size of the resulting terms, 3) only pathways with a significant *p*-value ≤ 0.05 passed the filter.

## Results

### Identification of rare variants in miRNAs from amyotrophic lateral sclerosis whole-genome data

To study the possible genetic contribution of miRNA variants to ALS pathogenesis and to predict their biological effects, we followed a three-step pipeline as shown in Figure 1. We firstly generated WGS data from a cohort of 100 sALS patients (Table 1). To obtain the dataset of miRNA genomic coordinates, we extracted all available information on discovered and published miRNAs from MiRBase. We retained a total of 505 miRNAs tagged by MiRBase as “high-confident”, which include miRNAs with a sufficient number of reads mapping both strands, 2 nucleotide-long 3’ overhangs, and a well-folded hairpin precursor (Kozomara and Griffiths-Jones, 2011). WGS data from our ALS cohort were merged and filtered to identify genetic variants in high-confident miRNA loci *via* VCFtools. Using this procedure, we successfully identified 159 variants out of 24,944,764 candidate sites. The majority of the identified variants were in a heterozygous state with only 15 in homozygosity. After annotation and filtering, 86 variants [82 single nucleotide variants (SNV), two insertions, and two deletions] in 79 sALS patients were classified as rare (MAF <0.01) according to both 1000 genomes and GnomAD databases. Of these, 6 (5 SNV and one deletion) were in homozygous state (Supplementary Table S1). The possible clinical significance of these variants was first evaluated using ClinVar and InterVar databases. As expected, none of them had already been reported or classified, except for two variants in the same miR-96 (chr7:g.129414568:G>A and chr7:g.129414574:A>G, GRCh37/hg19), which had already been identified as likely-benign and benign, respectively (Supplementary Table S1). The 86 identified rare variants mapped to 77 different miRNAs, mostly including a single variant each, with the exception of 11 miRNAs that harbored multiple rare variants (Supplementary Table S2). The miRNA expression profile across human tissues was also evaluated using the GTExportal. Expression data were available only for 12 (15.5%) out of the 77 identified miRNAs (Supplementary Figure S1) and miR-219a-2 showed a specific expression in the brain with a very high expression level in spinal cord (Table 2 and Supplementary Figure S1). Of interest, most of them were reported to be expressed in the cerebellum (10/12) or in the cortex (4/12), although with different expression values (Table 2).

**TABLE 2:** miRNA gene expression in brain areas according to GTEX database.

miRNA	Cortex	Cerebellum	Spinal cord
miR-219a-2	16.64	7.53	102.9
miR-324	0.89	2.68	0.37
miR-221	0.24	—	—
miR-590	—	0.77	—
miR-210	—	0.53	—
miR-181c	—	0.36	—
miR-199b	—	0.76	—
miR-339	0.28	0.77	—
miR-3613	—	0.37	—
miR-421	—	0.59	—

Expression values refer to “transcripts per million” (TPM) as reported in GTEx.

### *In silico* analyses of miRNA secondary structures

**FIGURE 2:**
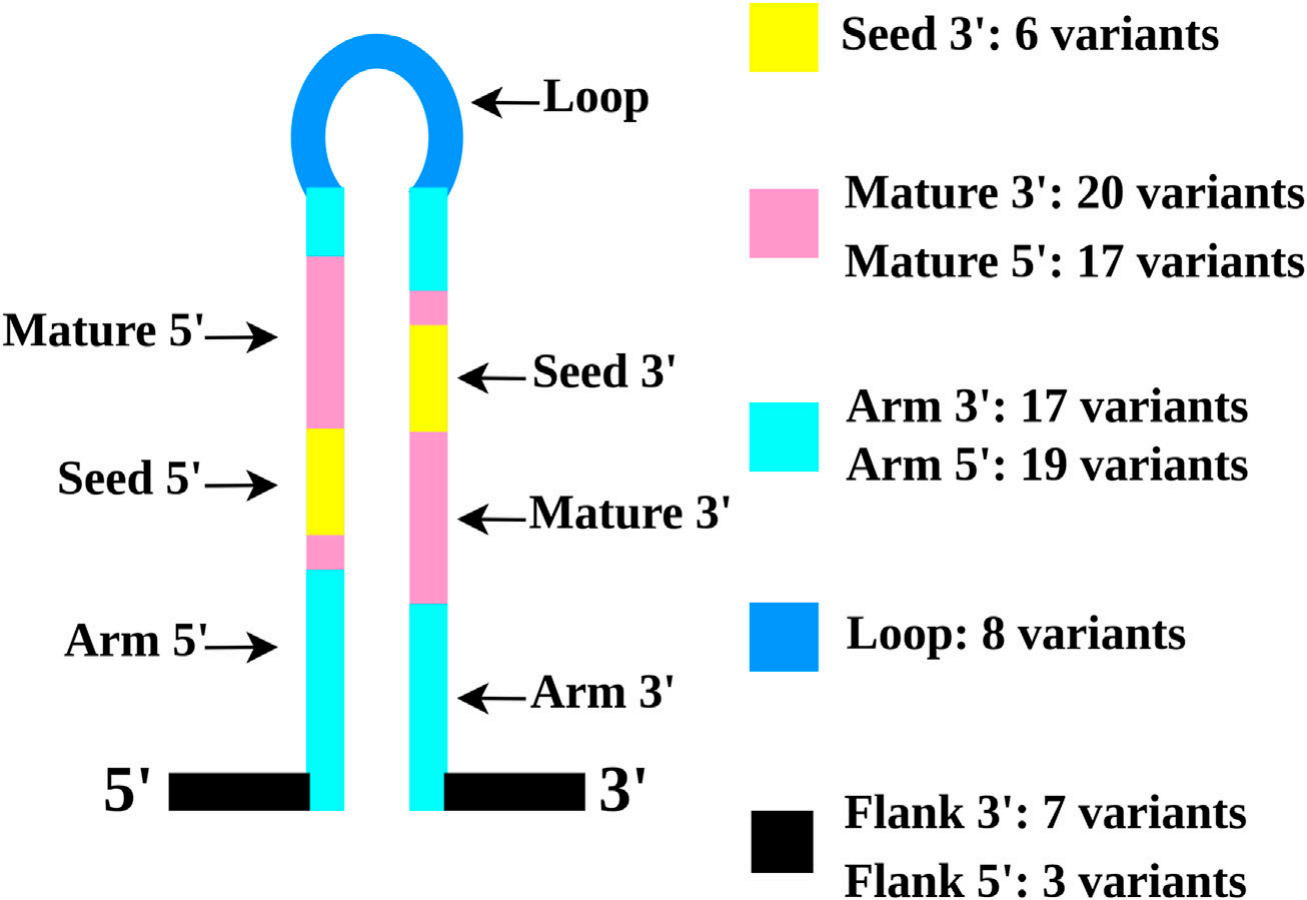
Schematic representation of ALS rare variants localization in miRNA precursors. The distinct miRNA regions are indicated by specific colors: seed (yellow), mature (pink), arms (light blue), loops (blue), and flanking region (black).

To assess the possible biological impact of the identified variants, we first established their localization in miRNA precursor secondary structures using miRVaS tool. The analysis predicted 97 different RNA conformations with variants located in different parts of the immature miRNA sequence (Supplementary Table S2). In particular, we obtained 10 RNA conformations with variants located in miRNA 5′ and 3′ flanking regions, 36 in the arm regions, eight in the loop regions, 37 in the mature regions, and 6 in the 3’ seed sequences (Figure 2).

**FIGURE 3:**
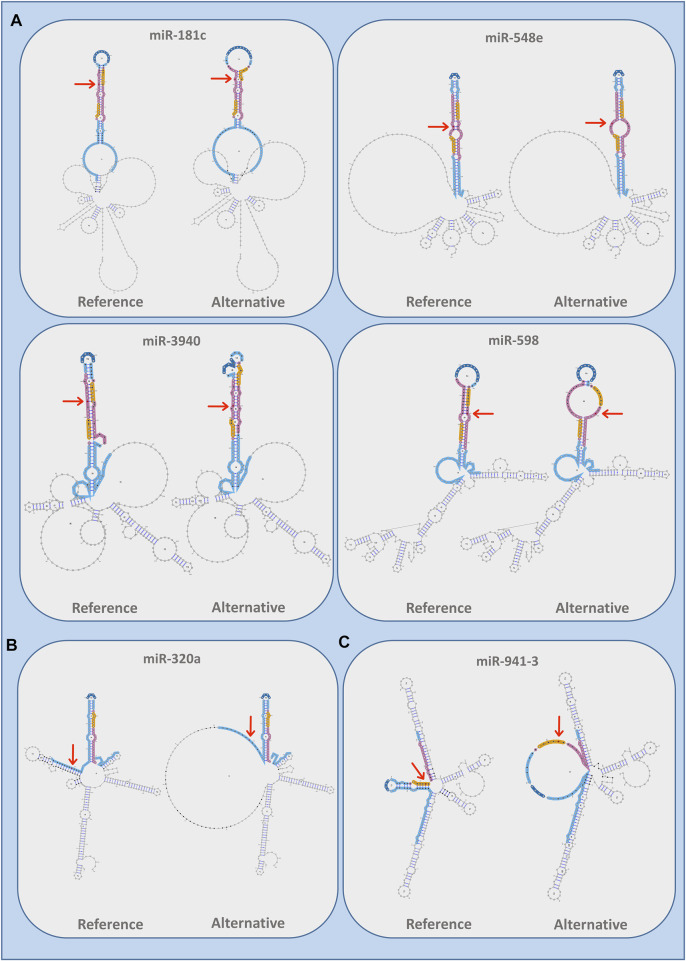
Images of RNA secondary structures remodeled by the presence of the rare variants in different miRNA regions as obtained by miRVaS using the centroid paradigm. **(A)** Variants located in mature regions of miR-181c (chr19:g13985554:G>A), miR-3940 (chr19:g.6416489:G>A), miR-548e (chr10:g.112748708:C>T), and miR-598 (chr8:g.10892743:C>T); **(B)** Variant located in arm domain of miR-320a (chr8:g.22102548:G>T); **(C)** Variant located in the seed sequence of miR-941-3 (chr20:g.62550965:G>A). The variant is indicated in red by an arrow; miRNA regions are indicated by specific colors: seed (yellow), mature (pink), arms (light blue), loops (blue), and flanking region (black).

We also evaluated whether ALS-associated variants had an impact on miRNA secondary structure according to all the three different paradigms used by miRVaS program, such as centroid, minimum free energy (MFE), and maximal expected accuracy (MEA). The majority of the predicted secondary RNA structures (71% for centroid, 73% for MEA, and 63% for MFE models) were changed compared to the wild-type reference sequence (Supplementary Table S2). More specifically, our analysis showed that a large part of variants modified the predicted conformation of the alternative miRNA at the level of seed and mature regions, which altogether account for 41% of predicted secondary structures (Table 3). Conversely, 30% of predictions showed no changes in miRNA secondary conformation due to the presence of the alternative ALS variant and none of the identified variants changed the secondary structure of the loop, a region important for pre-miRNA cleavage and maturation (Table 3). Intriguingly, the reference miRNA hairpin conformation appeared to be remodeled by substituting the wild-type sequence with the alternative one, independently on the variant localization in the mature (Figure 3A), arm (Figure 3B) or seed (Figure 3C) sequence of the miRNA (Supplementary Table S2).

**TABLE 3:** Analysis of structural impacts of miRNA variants by miRVaS.

Structural impact of the variant	Centroid (n° of predictions)	MEA (n° of predictions)	MFE (n° of predictions)	Total mean (%)
Arm	23	25	21	24
Flank	2	7	5	5
Mature	22	19	15	20
Seed	22	20	20	21
No change	28	26	36	30
Loop	—	—	—	0

Analyses were performed according to three different algorithms: Centroid, MEA (Maximum Expected Accuracy) and MFE (Minimum Free energy).

### Prediction of miRNA target genes

Since the region directly involved in target gene recognition is the 3′-seed sequence, we then focused our analysis on the 6 miRNAs carrying variants in the seed region, which include five SNV and a 3-nucleotide insertion (Table 4). Firstly, both the reference and the alternative 7-nucleotide long seed sequences were recruited from miRVaS. Potential target genes were then predicted using the Human TargetScan software. Both reference and alternative seed sequences were compared against the database of UTR sequences provided by the tool. Lists of target genes were finally generated for each reference (Supplementary Table S3) and alternative miRNA (Supplementary Table S4) seed sequence. This analysis showed that the number of potential targets for all mutated miRNAs changed and generally increased compared to the reference wild-type counterpart, except for miR-214 (chr1:g172107971:C>T) (9075 vs. 10149 target genes for the reference miRNA) (Table 5). However, when we specifically considered the novel predicted gene targets compared to the ones in common with the wild-type miRNA, also for the alternative miR-214 30% of the predicted targets were new and unique (Table 5). The proportion of novel targets ranged from 30% in miR-214 to 82% in miR-518a-1 (chr19:g.54234315:C>G), the latter reaching also the highest number of new potential targets (7,370 out of 8,891 total ones) (Table 5).

**TABLE 4:** List of miRNAs with rare variants in the seed sequence.

miRNA	Chr	Start	End	Variant	Ref	Alt	Ref_seed	Alt_seed
miR-214	1	172107970	172107971	snv	C	T	CAGCAGG	CAGCAAG
miR-4423	1	85599525	85599526	snv	T	C	UAGGCAC	CAGGCAC
miR-509-1	X	146342085	146342085	ins	—	ATC	GAUUGGU	GAUGAUU
miR-518a-1	19	54234314	54234315	snv	C	G	AAAGCGC	AAAGGGC
miR-5586	14	60113696	60113697	snv	C	T	AGAGUGA	AGAAUGA
miR-941-3	20	62550964	62550965	snv	G	A	ACCCGGC	ACCCAGC

Genome assembly GRCh37/hg19; Chr, chromosome; snv, single nucleotide variant; ins, insertion; Ref, reference sequence; Alt, alternative sequence; Ref_seed and Alt_seed, mature miRNA, seed sequence in the two conditions (the reference and alternative variants are indicated in red).

**TABLE 5:** Prediction of target genes of miRNAs with variants in the seed sequence.

miRNA	Ref seed gene targets	Alt seed gene targets	Common targets	New targets	ALS panel targets* (ref sequence)	ALS panel targets*	Common ALS targets	New ALS targets
	(n)	(n)	(n)	(n, and %)	(n)	(alt seed) (n)	(n)	(n, and %)
miR-214	10149	9075	6321	2754 (30%)	162	156	112	44 (28%)
miR-4423	5950	10031	4310	5721 (57%)	93	155	67	88 (56%)
miR-509-1	4008	5560	1912	3648 (65%)	63	98	35	63 (64%)
miR-518a-1	2207	8891	1521	7370 (82%)	40	167	30	137 (82%)
miR-5586	7264	8687	4702	3985 (45%)	124	148	87	61 (41%)
miR-941-3	2803	8806	2036	6770 (76%)	39	133	32	101 (75%)

*The ALS, panel includes 295 candidate genes previously reported (Eitan et al., 2022).

ref, reference miRNA, seed sequence; alt, alternative miRNA, seed sequence identified in our sALS, cohort.

We also crossed the predicted targets with a dataset of 295 candidate genes previously selected as likely associated with ALS disease (Eitan et al., 2022). Also in this case the proportion of the ALS-associated gene targets increased for all the miRNAs with the alternative seed variant compared to the reference sequence, again with the exception of miR-214 for which the number of ALS-specific targets remained nearly the same (156 in the mutant vs. 162 in the reference one) (Table 5). However, when considering only the ALS gene targets specifically predicted for the alternative seed sequence miRNAs, the proportion of unique ALS targets ranged from 28% for miR-214 to 82% for miR-518a-1 which again showed the highest number of unique ALS-associated targets (137 out 167 total ones) (Table 5).

**FIGURE 4:**
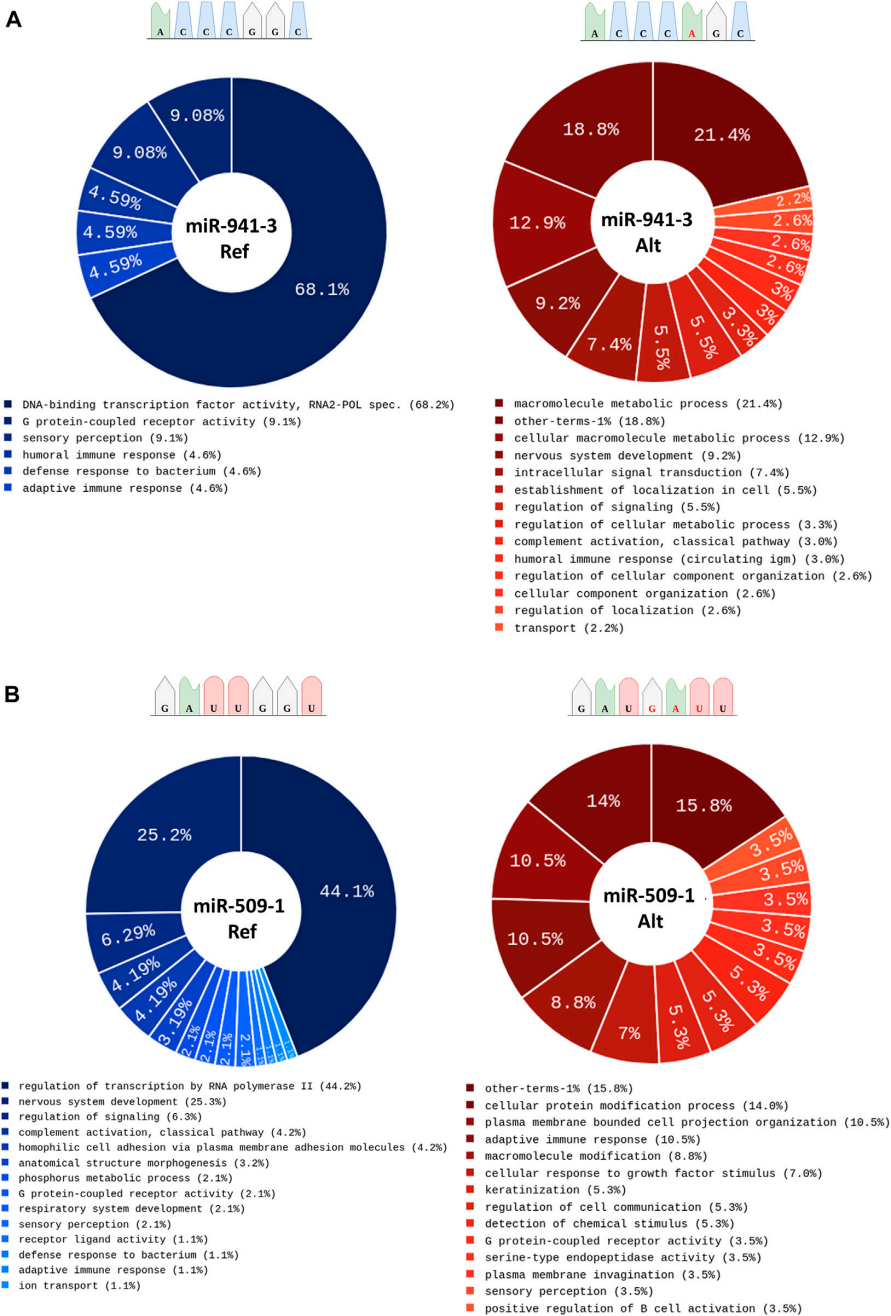
Over Representation Analysis performed on target genes predicted for the reference and the alternative variant in the miRNA seed sequence for **(A)** miR-941-3 and **(B)** miR-509-1.

Finally, to assess the possible impact of variants in miRNA seed sequence at biological level, we conducted an over-representation analysis on each dataset of predicted target genes *via* Cytoscape-ClueGo (Supplementary Table S5). Of interest, for the miR-941-3 alternative seed sequence (chr20:g.62550965:G>A), we found a change in the GO terms and the specific appearance of terms related to “nervous system development” compared to the reference seed sequence targets (Figure 4A). Conversely, for the ALS variant mapping in the seed sequence of miR-509-1 (chrX:g146342085:->ATC), we observed the opposite condition with the disappearance of the GO term related to “nervous system development” compared to the reference sequence target genes (Figure 4B). Regarding the other four miRNAs, we found an enrichment in GO terms mostly in common with the reference miRNA sequence, in line with the increased number of predicted target genes for the mutant seed variants (Supplementary Table S5).

## Discussion

The contribution of noncoding variants to complex disease etiology is still largely unknown although WGS data are now available for most multifactorial disorders. Genetic analyses are mainly focused on the identification of rare coding variants in order to identify possible risk factors associated with disease onset and/or survival. However, noncoding variants may act as important modifying factors, at both transcriptional and post-transcriptional levels. Not only promoter and 5′UTR sequences influence transcription, but also variants in enhancers and topologically associated domains, as emerging from 3D chromatin conformation studies. At post-transcriptional level, intronic variants affect splicing, including that of cryptic exons, as well as noncoding variants in 3′UTR change binding of miRNAs and RNA-binding proteins, therefore influencing mRNA transport, translation, and decay. In this context, analysis of genetic variants in non-coding elements such as miRNAs and long noncoding RNAs (lncRNAs) has so far received poor attention in the field of complex diseases, while mutations in miRNAs proved to be causative of some hereditary disorders, such as spondyloepiphyseal dysplasia (Grigelioniene et al., 2019) and nonsyndromic hearing loss (Mencía et al., 2009).

Here we developed a pipeline to identify and study rare variants in miRNA sequences from WGS data of sALS patients, that may affect their folding and secondary structures and, likely, their proper biogenesis as well as their target gene recognition. For our analysis, we selected only those miRNAs with the highest probability of being annotated as miRNAs in miRBase and obtained 505 miRNAs out of the 1750 ones present in the database. Our analysis defined a set of 86 noncoding and rare (MAF <0.01) variants mapping in distinct regions of 77 pre-miRNAs. The localization of these variants in miRNA precursors and the impact of these variants on their folding were evaluated using miRVaS which is, currently, the only tool available and conceived for this purpose (Lukasik et al., 2016). Predictions were performed using default parameters and including 100 nucleotides surrounding the hairpin as suggested by miRVaS (Cammaerts et al., 2016) because also variants mapping outside the miRNA hairpin may have a functional effect (Chen et al., 2004). We successfully identified multiple RNA secondary structures changed by the presence of the ALS-associated variants in miRNA sequences. These structural modifications may alter recognition and cleavage by DROSHA or DICER, the two processing enzymes required for the maturation of miRNA precursors, and potentially lead to a defective miRNA biogenesis with effects also on target mRNA translation. As regards DROSHA, it was already demonstrated that the presence of point variants and mismatches in the hairpin could affect miRNA processing by changing RNA secondary structures, a molecular mechanism used to finely regulate the expression of specific miRNAs during cell differentiation (Sperber et al., 2014). In human ALS post-mortem motoneurons, miR-218-2 expression is significantly downregulated and, in a genetic screening of a large ALS cohort, the presence of 6 rare variants in miR-218-2 was functionally associated with its defective processing by DICER (Reichenstein et al., 2019). The decreased biogenesis of miR-218-2 determined, as a consequence, the upregulation of its target genes, including the potassium channel Kv10.1, and induced dysregulation of neuronal activity as a possible ALS pathomechanism (Reichenstein et al., 2019). Moreover, the cleavage activity of DICER on pre-miRNAs was shown to strongly depend on the presence of specific single-nucleotide bulges in their secondary structure, which can therefore have a role in controlling miRNA biogenesis (Nguyen et al., 2022). Thus, by considering all these observations and our *in silico* predictions, we speculate that the gene variants we found in miR-598 and miR-941-3, by changing the hairpin RNA conformation, might determine a defective cleavage activity by DROSHA. Conversely, the identified variant in miR-3940 might impair its proper maturation by DICER because of the formation of a bulge in its secondary structure.

Our analysis also identified rare variants in the 3′-seed sequences of 6 miRNAs, which represent the functional region of these small non-coding RNAs. The recognition and binding of target genes are, in fact, due to the perfect matching with the miRNA seed sequence. Therefore, variants in this 7-nucleotide long sequence are supposed to exert a biological effect by changing the set of recognized and bound target genes. Our *in silico* predictions do confirm that all the 6 miRNAs harbouring a variant in the seed sequence show a very different array of targets compared to their reference miRNAs, also when considering a specific subset of ALS-associated genes (Eitan et al., 2022). Of interest, by conducting an ORA of the newly identified target genes for the seed-mutated miRNAs, we found changes in the GO pattern, especially in GO terms related to nervous system development for the mutant miR-941-3 and miR-509-1 target genes. Our findings clearly suggest that ALS-associated mutations in miRNA seed sequence may gain a function in regulating novel genes and different cell pathways.

A critical point in trying to define the biological effects of miRNA genetic variants is also represented by the scarce availability of expression data about miRNAs, about which we obtained information only for 15% of them, although most of them (10/12) showed expression in neuronal tissues. In particular, one miRNA (miR-219a-2) showed a very specific expression in the brain with the highest expression level in spinal cord. However, few available literature data only report that miR-219a-2 is up-regulated in the synaptosome fraction obtained from post-mortem brains of patients suffering from major depressive disorder (Yoshino et al., 2021).

Another critical point is represented by the need to validate *in silico* predictions by functional *in vitro*/*in vivo* assays. Establishing the real impact and a clear relevance of these miRNA gene variants on ALS onset and disease progression therefore remains a burdensome task. So far, the majority of studies on ALS have indeed focused on the identification of miRNAs as possible prognostic factors whose expression is altered in patients’ biofluids, including miR-124, miR-155, miR-181a1/b1, miR-181a2/b2, and miR-206 (Toivonen et al., 2014; Cunha et al., 2018, 155; Magen et al., 2021; Vaz et al., 2021; Banack et al., 2022; Joilin et al., 2022). In our analysis, no rare variants were found in these specific candidate miRNA loci, likely due to our small sample size. However, the association between ALS phenotype and noncoding regulatory sequences has been recently tested using several thousands of WGS data from ProjectMinE sequencing Consortium. Rare variants in 1750 autosomal miRNA genes and in 295 noncoding 3′UTR of candidate genes linked to sALS were aggregated to test the relationship with the disease (Eitan et al., 2022). No significant associations emerged from these data, possibly because of the small size of miRNA genes which makes this analysis particularly complex at statistical level.

Despite the lack of conclusive functional data, we here provide an operative workflow to analyze miRNA genetic variability from WGS data and to predict the biological effects of non-coding variants on pre-miRNA folding and on miRNA target gene recognition. This pipeline could be adopted for future studies on larger WGS datasets regarding not only ALS but, more broadly, all complex diseases.

## Data Availability

The dataset presented in this study can be found in the online repository ENA (European Nucleotide Archive) with the study accession number PRJEB57326.

## References

[B1] Agarwal V. Bell G. W. Nam J.-W. Bartel D. P. (2015). Predicting effective microRNA target sites in mammalian mRNAs. eLife 4, e05005. 10.7554/eLife.05005 26267216 PMC4532895

[B2] Alvia M. Aytan N. Spencer K. R. Foster Z. W. Rauf N. A. Guilderson L. (2022). MicroRNA alterations in chronic traumatic encephalopathy and amyotrophic lateral sclerosis. Front. Neurosci. 16, 855096. 10.3389/fnins.2022.855096 35663558 PMC9160996

[B3] Banack S. A. Dunlop R. A. Stommel E. W. Mehta P. Cox P. A. (2022). miRNA extracted from extracellular vesicles is a robust biomarker of amyotrophic lateral sclerosis. J. Neurol. Sci. 442, 120396. 10.1016/j.jns.2022.120396 36081303

[B4] Bindea G. Mlecnik B. Hackl H. Charoentong P. Tosolini M. Kirilovsky A. (2009). ClueGO: A Cytoscape plug-in to decipher functionally grouped gene ontology and pathway annotation networks. Bioinformatics 25, 1091–1093. 10.1093/bioinformatics/btp101 19237447 PMC2666812

[B5] Brooks B. R. Miller R. G. Swash M. Munsat T. L. (2000). El Escorial revisited: Revised criteria for the diagnosis of amyotrophic lateral sclerosis. Amyotroph. Lateral Scler. Other Mot. Neuron Disord. 1, 293–299. 10.1080/146608200300079536 11464847

[B6] Cammaerts S. Strazisar M. Dierckx J. Del Favero J. De Rijk P. (2016). miRVaS: a tool to predict the impact of genetic variants on miRNAs. Nucleic Acids Res. 44, e23. 10.1093/nar/gkv921 26384425 PMC4756848

[B7] Chen C.-Z. Li L. Lodish H. F. Bartel D. P. (2004). MicroRNAs modulate hematopoietic lineage differentiation. Science 303, 83–86. 10.1126/science.1091903 14657504

[B8] Cunha C. Santos C. Gomes C. Fernandes A. Correia A. M. Sebastião A. M. (2018). Downregulated glia interplay and increased miRNA-155 as promising markers to track ALS at an early stage. Mol. Neurobiol. 55, 4207–4224. 10.1007/s12035-017-0631-2 28612258

[B9] Danecek P. Auton A. Abecasis G. Albers C. A. Banks E. DePristo M. A. (2011). The variant call format and VCFtools. Bioinformatics 27, 2156–2158. 10.1093/bioinformatics/btr330 21653522 PMC3137218

[B10] Darty K. Denise A. Ponty Y. (2009). Varna: Interactive drawing and editing of the RNA secondary structure. Bioinformatics 25, 1974–1975. 10.1093/bioinformatics/btp250 19398448 PMC2712331

[B11] de Carvalho J. B. de Morais G. L. Vieira T. C. dos S. Rabelo N. C. Llerena J. C. (2019). miRNA genetic variants alter their secondary structure and expression in patients with RASopathies syndromes. Front. Genet. 10, 1144. 10.3389/fgene.2019.01144 31798637 PMC6863982

[B12] DePristo M. A. Banks E. Poplin R. E. Garimella K. V. Maguire J. R. Hartl C. (2011). A framework for variation discovery and genotyping using next-generation DNA sequencing data. Nat. Genet. 43, 491–498. 10.1038/ng.806 21478889 PMC3083463

[B13] Devuyst O. (2015). The 1000 genomes Project: Welcome to a new world. Perit. Dial. Int. 35, 676–677. 10.3747/pdi.2015.00261 26703842 PMC4690620

[B14] Di Pietro L. Lattanzi W. Bernardini C. (2018). Skeletal muscle MicroRNAs as key players in the pathogenesis of amyotrophic lateral sclerosis. Int. J. Mol. Sci. 19, 1534. 10.3390/ijms19051534 29786645 PMC5983603

[B15] Duan J. Shi J. Fiorentino A. Leites C. Chen X. Moy W. (2014). A rare functional noncoding variant at the GWAS-implicated MIR137/MIR2682 locus might confer risk to schizophrenia and bipolar disorder. Am. J. Hum. Genet. 95, 744–753. 10.1016/j.ajhg.2014.11.001 25434007 PMC4259974

[B16] Eitan C. Siany A. Barkan E. Olender T. van Eijk K. R. Moisse M. (2022). Whole-genome sequencing reveals that variants in the interleukin 18 receptor accessory protein 3′UTR protect against ALS. Nat. Neurosci. 25, 433–445. 10.1038/s41593-022-01040-6 35361972 PMC7614916

[B17] Goutman S. A. Hardiman O. Al-Chalabi A. Chió A. Savelieff M. G. Kiernan M. C. (2022). Emerging insights into the complex genetics and pathophysiology of amyotrophic lateral sclerosis. Lancet. Neurol. 21, 465–479. 10.1016/S1474-4422(21)00414-2 35334234 PMC9513754

[B18] Grigelioniene G. Suzuki H. I. Taylan F. Mirzamohammadi F. Borochowitz Z. U. Ayturk U. M. (2019). Gain-of-function mutation of microRNA-140 in human skeletal dysplasia. Nat. Med. 25, 583–590. 10.1038/s41591-019-0353-2 30804514 PMC6622181

[B19] Hardiman O. Al-Chalabi A. Chio A. Corr E. M. Logroscino G. Robberecht W. (2017). Amyotrophic lateral sclerosis. Nat. Rev. Dis. Prim. 3, 17071–17119. 10.1038/nrdp.2017.71 28980624

[B20] Hop P. J. Zwamborn R. A. J. Hannon E. Shireby G. L. Nabais M. F. Walker E. M. (2022). Genome-wide study of DNA methylation shows alterations in metabolic, inflammatory, and cholesterol pathways in ALS. Sci. Transl. Med. 14, eabj0264. 10.1126/scitranslmed.abj0264 35196023 PMC10040186

[B21] Joilin G. Gray E. Thompson A. G. Talbot K. Leigh P. N. Newbury S. F. (2022). Profiling non-coding RNA expression in cerebrospinal fluid of amyotrophic lateral sclerosis patients. Ann. Med. 54, 3069–3078. 10.1080/07853890.2022.2138530 36314539 PMC9629092

[B22] Joilin G. Leigh P. N. Newbury S. F. Hafezparast M. (2019). An overview of MicroRNAs as biomarkers of ALS. Front. Neurol. 10, 186. 10.3389/fneur.2019.00186 30899244 PMC6416171

[B23] Juźwik C. A. Drake S. Zhang Y. Paradis-Isler N. Sylvester A. Amar-Zifkin A. (2019). microRNA dysregulation in neurodegenerative diseases: A systematic review. Prog. Neurobiol. 182, 101664. 10.1016/j.pneurobio.2019.101664 31356849

[B24] Karczewski K. J. Francioli L. C. Tiao G. Cummings B. B. Alföldi J. Wang Q. (2020). The mutational constraint spectrum quantified from variation in 141, 456 humans. Nature 581, 434–443. 10.1038/s41586-020-2308-7 32461654 PMC7334197

[B25] Kim K. Y. Kim Y. R. Choi K. W. Lee M. Lee S. Im W. (2020). Downregulated miR-18b-5p triggers apoptosis by inhibition of calcium signaling and neuronal cell differentiation in transgenic SOD1 (G93A) mice and SOD1 (G17S and G86S) ALS patients. Transl. Neurodegener. 9, 23. 10.1186/s40035-020-00203-4 32605607 PMC7328278

[B26] Kozomara A. Birgaoanu M. Griffiths-Jones S. (2019). miRBase: from microRNA sequences to function. Nucleic Acids Res. 47, D155–D162. 10.1093/nar/gky1141 30423142 PMC6323917

[B27] Kozomara A. Griffiths-Jones S. (2011). miRBase: integrating microRNA annotation and deep-sequencing data. Nucleic Acids Res. 39, D152–D157. 10.1093/nar/gkq1027 21037258 PMC3013655

[B28] Li H. Durbin R. (2009). Fast and accurate short read alignment with Burrows–Wheeler transform. Bioinformatics 25, 1754–1760. 10.1093/bioinformatics/btp324 19451168 PMC2705234

[B29] Lorenz R. Bernhart S. H. Höner zu Siederdissen C. Tafer H. Flamm C. Stadler P. F. (2011). ViennaRNA package 2.0. Algorithms Mol. Biol. 6, 26. 10.1186/1748-7188-6-26 22115189 PMC3319429

[B30] Lukasik A. Wójcikowski M. Zielenkiewicz P. (2016). Tools4miRs – one place to gather all the tools for miRNA analysis. Bioinformatics 32, 2722–2724. 10.1093/bioinformatics/btw189 27153626 PMC5013900

[B31] Magen I. Yacovzada N. S. Yanowski E. Coenen-Stass A. Grosskreutz J. Lu C.-H. (2021). Circulating miR-181 is a prognostic biomarker for amyotrophic lateral sclerosis. Nat. Neurosci. 24, 1534–1541. 10.1038/s41593-021-00936-z 34711961

[B32] McKenna A. Hanna M. Banks E. Sivachenko A. Cibulskis K. Kernytsky A. (2010). The genome analysis toolkit: A MapReduce framework for analyzing next-generation DNA sequencing data. Genome Res. 20, 1297–1303. 10.1101/gr.107524.110 20644199 PMC2928508

[B33] Mencía A. Modamio-Høybjør S. Redshaw N. Morín M. Mayo-Merino F. Olavarrieta L. (2009). Mutations in the seed region of human miR-96 are responsible for nonsyndromic progressive hearing loss. Nat. Genet. 41, 609–613. 10.1038/ng.355 19363479

[B34] Nguyen T. D. Trinh T. A. Bao S. Nguyen T. A. (2022). Secondary structure RNA elements control the cleavage activity of DICER. Nat. Commun. 13, 2138. 10.1038/s41467-022-29822-3 35440644 PMC9018771

[B35] Panio A. Cava C. D’Antona S. Bertoli G. Porro D. (2022). Diagnostic circulating miRNAs in sporadic amyotrophic lateral sclerosis. Front. Med. 9, 861960. 10.3389/fmed.2022.861960 PMC9121628 35602517

[B36] Reichenstein I. Eitan C. Diaz-Garcia S. Haim G. Magen I. Siany A. (2019). Human genetics and neuropathology suggest a link between miR-218 and amyotrophic lateral sclerosis pathophysiology. Sci. Transl. Med. 11, eaav5264. 10.1126/scitranslmed.aav5264 31852800 PMC7057809

[B37] Rinchetti P. Rizzuti M. Faravelli I. Corti S. (2018). MicroRNA metabolism and dysregulation in amyotrophic lateral sclerosis. Mol. Neurobiol. 55, 2617–2630. 10.1007/s12035-017-0537-z 28421535

[B38] Shannon P. Markiel A. Ozier O. Baliga N. S. Wang J. T. Ramage D. (2003). Cytoscape: A software environment for integrated models of biomolecular interaction networks. Genome Res. 13, 2498–2504. 10.1101/gr.1239303 14597658 PMC403769

[B39] Sperber H. Beem A. Shannon S. Jones R. Banik P. Chen Y. (2014). miRNA sensitivity to Drosha levels correlates with pre-miRNA secondary structure. RNA 20, 621–631. 10.1261/rna.043943.113 24677349 PMC3988564

[B40] Spielmann M. Mundlos S. (2016). Looking beyond the genes: The role of non-coding variants in human disease. Hum. Mol. Genet. 25, R157–R165. 10.1093/hmg/ddw205 27354350

[B41] Toivonen J. M. Manzano R. Oliván S. Zaragoza P. García-Redondo A. Osta R. (2014). MicroRNA-206: A potential circulating biomarker candidate for amyotrophic lateral sclerosis. PLOS ONE 9, e89065. 10.1371/journal.pone.0089065 24586506 PMC3930686

[B42] Van der Auwera G. A. Carneiro M. O. Hartl C. Poplin R. del Angel G. Levy-Moonshine A. (2013). From FastQ data to high confidence variant calls: The genome analysis toolkit best practices pipeline. Curr. Protoc. Bioinforma. 11, 11.10.1–11.10.33. 10.1002/0471250953.bi1110s43 PMC4243306 25431634

[B43] van Rheenen W. van der Spek R. A. A. Bakker M. K. van Vugt J. J. F. A. Hop P. J. Zwamborn R. A. J. (2021). Common and rare variant association analyses in amyotrophic lateral sclerosis identify 15 risk loci with distinct genetic architectures and neuron-specific biology. Nat. Genet. 53, 1636–1648. 10.1038/s41588-021-00973-1 34873335 PMC8648564

[B44] Vaz A. R. Vizinha D. Morais H. Colaço A. R. Loch-Neckel G. Barbosa M. (2021). Overexpression of miR-124 in motor neurons plays a key role in ALS pathological processes. Int. J. Mol. Sci. 22, 6128. 10.3390/ijms22116128 34200161 PMC8201298

[B45] Wang K. Li M. Hakonarson H. (2010). Annovar: Functional annotation of genetic variants from high-throughput sequencing data. Nucleic Acids Res. 38, e164. 10.1093/nar/gkq603 20601685 PMC2938201

[B46] Williams S. M. An J. Y. Edson J. Watts M. Murigneux V. Whitehouse A. J. O. (2019). An integrative analysis of non-coding regulatory DNA variations associated with autism spectrum disorder. Mol. Psychiatry 24, 1707–1719. 10.1038/s41380-018-0049-x 29703944

[B47] Yoshino Y. Roy B. Dwivedi Y. (2021). Differential and unique patterns of synaptic miRNA expression in dorsolateral prefrontal cortex of depressed subjects. Neuropsychopharmacology 46, 900–910. 10.1038/s41386-020-00861-y 32919404 PMC8115313

