# Supplementary material for: Analysis of miRNA rare variants in amyotrophic lateral sclerosis and in silico prediction of their biological effects
Source: Front Genet. 2022 Dec 7;13:1055313. doi: 10.3389/fgene.2022.1055313 (PMC9768194; doi:10.3389/fgene.2022.1055313)

SUPPLEMENTARY DATA

Figure S1. Gene expression profile of candidate miRNAs identified from ALS WGS data.

Expression data were obtained from the GTEX portal and 63 out of the 77 identified miRNAs were annotated in the database (https://gtexportal.org).

The heatmap shows the expression profile across tissues which was availble for only 12 miRNAs (15%; 12/77). Expression values are indicated as TPM (transcripts per million).

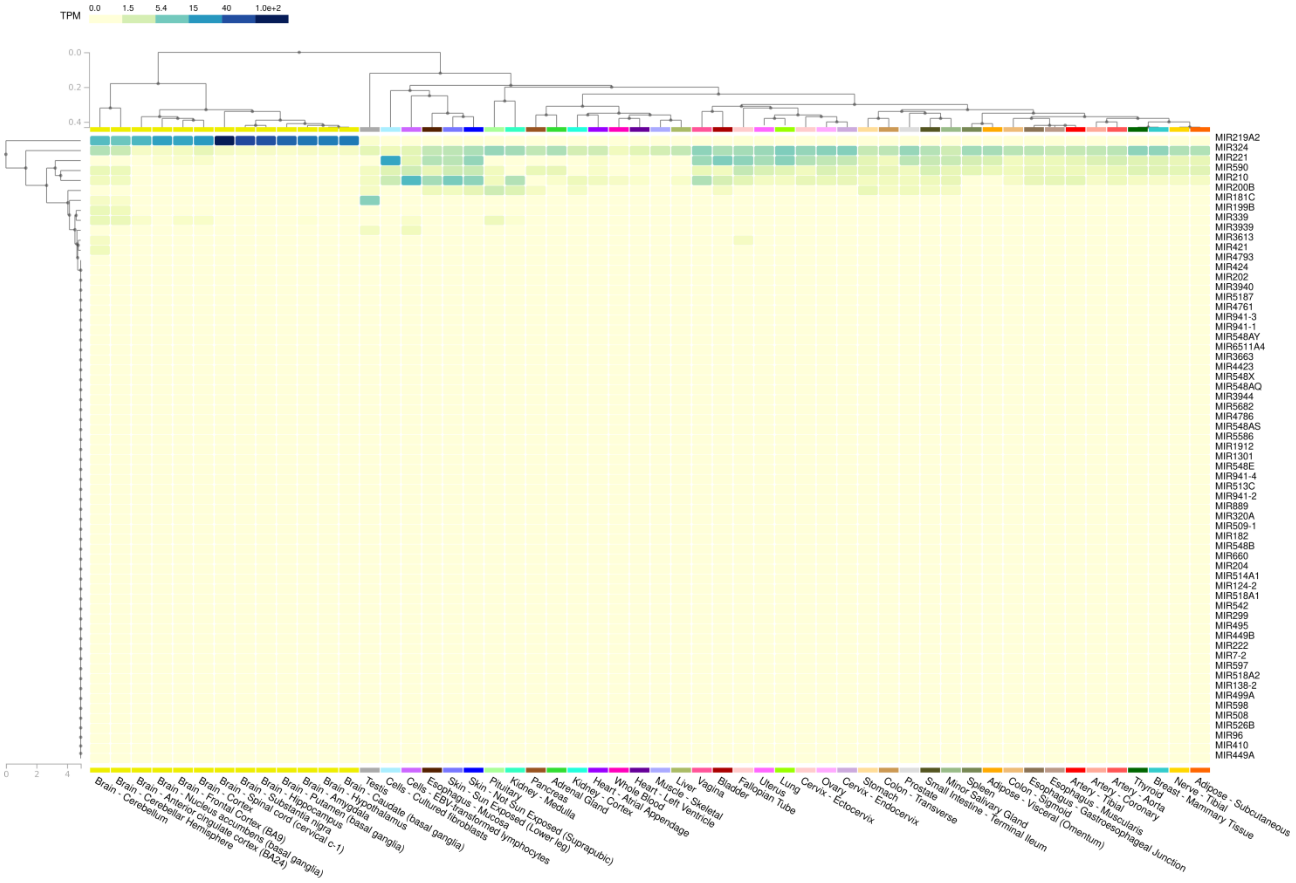

Supplement: Supplementary file 1 [file DataSheet1.PDF]
